# Reliability of using Wood’s lamp by shelter personnel to diagnose *Microsporum canis* in cats

**DOI:** 10.17221/32/2023-VETMED

**Published:** 2023-07-28

**Authors:** Karolina Mrazkova, Jarmila Konvalinova, Iveta Bedanova

**Affiliations:** Department of Animal Protection, Welfare and Ethology, Faculty of Veterinary Hygiene and Ecology, University of Veterinary Sciences Brno, Brno, Czech Republic

**Keywords:** culturing, dermatophyte, Sabouraud’s agar, skin disease

## Abstract

Optimising diagnostic methods in shelters so that they are as economical as possible for the shelter is especially important because shelters often have a significant lack of funds and so usually do not carry out preventive screening of cats. Dermatophyte fungi spread quickly and can infect shelter staff. The aim of our work was to identify the occurrence of *Microsporum canis* in shelter cats. It aimed to determine the prevalence of *M.* *canis* in cats at the selected shelter and compare the efficiency of detection using a Wood’s lamp and culturing on Sabouraud’s agar. All cats present in the shelter at the time of the study (*n* = 70) were examined with Wood’s lamp and hair sampling followed by subsequent culturing on Sabouraud’s agar. Identification of fungi was based on microscopic proof of macroconidia and microconidia. The prevalence of *M.* *canis* by diagnosis on Sabouraud’s agar was 64.29% of cats, with the help of Wood’s lamp 48.57% of cats showed positive fluorescence. The sensitivity of the Wood lamp examination was 71% and the specificity was 92%. Our study suggests that Wood’s lamp could be used by trained shelter personnel for the first examination of cats at reception and could significantly reduce the risk of spreading *M.* *canis* in shelters.

The issue of dermatophyte fungi occurrence in shelter cats is a current topic. Shelters often struggle with finances, staff, and assurance of quarantine for newly arriving animals. Moreover, the zoonotic potential of *M. canis* cannot be overlooked.

Cats in shelters are often in poor health condition and stressed. The presence of a large number of animals in one place also leads to easier spreading of infection. Examination of samples at veterinary clinics is often based on dermatophyte test medium (DTM) with subsequent fungi identification. Its financial demands and the need for daily checks can make it difficult to use in shelters. It consists of Sabouraud dextrose agar with cycloheximide, gentamicin, chlortetracycline, and phenol red. Cycloheximide is a substance that inhibits the growth of saprophytic fungi, chlortetracycline and gentamicin are antibiotics that inhibit the growth of bacteria on the medium. Phenol red serves as an indicator that depends on the change in pH (Horne et al. 2019). Metabolites are released during the growth of dermatophytes that alkalinise the pH and thus cause the colour of the medium to change from yellow to red. The colour change comes along with the growth of the colony. The colonies should grow within seven days from the inoculation of samples (Paterson 2017).

Another option is to culture the collected samples on Sabouraud’s agar (SA). This method is time-consuming and the animals would have to stay in quarantine for several weeks before the examination result would be known. However, the SA culture is often mentioned as one of the most reliable methods of dermatophyte fungi diagnostics, especially if performed in a specialised laboratory by knowledgeable staff. A significant disadvantage of the SA examination is the time required for cultivation (Moriello and Deboer 1991; Moskaluk and VandeWoude 2022). Samples are most often collected by grooming. They are incubated at the temperature of 23 °C as dermatophytes sporulate best at this temperature (Moriello 2014). Some authors indicate 25 °C as the right temperature (Santana et al. 2020). Direct microscopy can also be used to diagnose the dermatophyte fungi. The speed of diagnostics and thus the possibility of starting the therapy immediately are the advantages of direct microscopy (Hubka et al. 2018). It is difficult to identify the type of fungi involved in the development of the disease in a given patient by direct microscopy. Hyphae inside the hair and spores on its surface are visible during the examination under a microscope (Svoboda et al. 2008). An experienced examiner is also needed.

Another possibility for the diagnosis of dermatophyte fungi is the use of molecular genetic methods, especially polymerase chain reaction (PCR), but also (reverse transcription polymerase chain reaction (RT-PCR) or possibly more sophisticated methods, such as polymerase chain reaction-enzyme-linked immunosorbent assay (PCR-ELISA), polymerase chain reaction reverse-line-blotting (PCR-RLB), and microarrays (Hubka et al. 2018). PCR can be very helpful in the detection of dermatophytes. However, a positive PCR result does not necessarily mean an active infection, as dead parts of fungi can persist in the coat and can be detected by PCR even after successful treatment; furthermore, PCR can be positive also in animals that act as mechanical carriers of spores (Moriello et al. 2017; Hubka et al. 2018). This method cannot be used for treatment monitoring in a short period of time, however, a negative PCR after the treatment proves its success (Hubka et al. 2018). The method has been recently developed and diagnostics can also be performed from paraffin blocks (Hubka et al. 2018). Matrix-assisted laser desorption/ionization in combination with a time-of-flight detector can also be used to diagnose dermatophyte fungi. This is a method that has been highly successful in veterinary microbiology laboratories (Dhiman et al. 2011). Compared to conventional dermatophyte diagnostic methods, this method is much faster, more efficient, and more straightforward. However, the implementation of this method is somewhat difficult in practice. Microscopic filamentous fungi are biologically quite complex organisms, they grow slowly and often produce pigments. Moreover, there is no clear definition of species for some taxa. However, the implementation of this method is undoubtedly a major advance in mycological diagnostics and an interesting alternative to other more laborious methods (Gnat et al. 2020).

Examination with Wood’s lamp appears to be very useful for *M.* *canis* detection in shelter cats. Wood’s lamp is a diagnostic instrument that uses UV radiation with a wavelength from 320 nm to 400 nm, but most often 365 nm. It was invented by physicist Robert W. Wood as a communication tool in World War I (Moriello et al. 2017). Infested yellow-green fluorescent hair is suitable for sampling for microscopy and culture examination in practice. A substance called pteridine is responsible for the fluorescence of the infested hair (Moriello et al. 2017). When using a Wood lamp, the lamp must first be allowed to heat up for several minutes (Taylor 2010). The examination using the Wood’s lamp has a relatively high predictive value, however, the results should be always confirmed microscopically or by culture (Hubka et al. 2018).

Wood’s lamp examination is cheap and easy, so it seems suitable for use in shelters that do not have enough funds and therefore do not perform a culture examination of all newly admitted animals. The use of Wood’s lamp by well-trained shelter personnel could minimise the occurrence of *M.* *canis* infection in shelters.

## MATERIAL AND METHODS

Seventy cats from a private shelter were examined in October 2021. The age, sex, and health condition of the cats were monitored.

The cats were divided into three age categories: kittens (0 to 1 year), adults (1 to 8 years), and seniors (8+). The cats were further divided into one group showing clinical symptoms of skin disease and another group without such symptoms. The group of cats showing clinical symptoms included 38 individuals in total whereas the group of cats without clinical symptoms included 32 cats. All cats were examined using a Wood’s lamp and the collected samples were cultured on Sabouraud’s agar. Examination using Wood’s lamp took place in a darkened room. The Wood’s lamp was left to warm up for 5 min before use. During the examination, the lamp was held approximately 5 cm from the body surface. Green-yellow fluorescence was monitored. Samples for culture examination were collected by grooming the hair with a sterile toothbrush. A new toothbrush was used for each cat. The collected hair was transferred into Petri dishes and transported to the laboratory. The samples were plated on Sabouraud agar in the laboratory and cultured at 25 °C for 21 days. The fungi were identified by microscopic examination of the colonies. The microscopic examination was performed using a 40 × magnification. The results were processed in Excel in the form of tables. Statistical processing was performed in UNISTAT v6.5 software. Differences in frequencies were tested using the Chi-square test within the 2 × 2 contingency table methodology. Values smaller than 0.05 were considered significant and values smaller than 0.01 were considered to be highly significant.

## RESULTS

*Microsporum canis* was identified by culture in 45 cats (64.29%). The Wood’s lamp examination showed a positive fluorescence in 34 cats (48.57%), see Table 1.

**Table 1:** Comparison of Wood’s lamp test and culture in all examined cats

Examination method	Positive	%	Negative	%	Total	Significance
Wood’s lamp	34	48.57	36	51.43	70	*P =* 0.088
Culture	45	64.29	25	35.71	70	

In the group of kittens (*n* = 29), the culture was positive at 65.52% of cases (*n* = 19). Adult cats (*n* = 36) were positive in 63.89% of cases (*n* = 23). In the senior group (*n* = 5), 60% of cases (*n* = 3) were positive, see Table 2. In the category of kittens, positive fluorescence appeared in 55.17% (*n* = 16) of cats, while the adult category showed positive fluorescence in 44.44% (*n* = 6) of cats. In the senior category, 20% (*n* = 1) of cats showed positive fluorescence (Table 3).

**Table 2:** Culture results in individual age categories of cats

Age category	Culture (+)	%	Culture (–)	%
Kittens	19	65.52	10	34.48
Adults	23	63.89	13	36.11
Seniors	3	60.00	2	40.00

**Table 3:** Results of Wood’s lamp examination in individual age categories of cats

Age category	Wood’s lamp (+)	%	Wood’s lamp (–)	%
Kittens	16	55.17	13	44.83
Adults	16	44.44	20	55.56
Seniors	1	20.00	4	80.00

A total of 89.47% (*n* = 34) of cats showing clinical symptoms were positive, whereas, in the category without clinical symptoms, 34.38% (*n* = 11) were positive. In the category of cats with symptoms, 81.58% (*n* = 31) of cats showed positive fluorescence during the Wood’s lamp examination, while in the category without clinical symptoms, 6.25% (*n* = 2) cats showed positive fluorescence (Tables 4 and 5).

**Table 4:** Comparison of Wood’s lamp and culture in cats with clinical symptoms

Examination method	Positive	%	Negative	%	Total	Significance
Wood’s lamp	31	81.58	7	18.42	38	*P =* 0.163
Culture	34	89.47	4	10.53	38	

**Table 5:** Comparison of Wood’s lamp and culture in cats without clinical symptoms

Examination method	Positive	%	Negative	%	Total	Significance
Wood’s lamp	2	6.25	30	93.75	32	*P =* 0.005
Culture	11	34.38	21	65.63	32	

The statistical comparison did not show any significant difference in the diagnosis of dermatophytic fungi with Wood’s lamp, or culture (*P* = 0.088), and there was no statistically significant difference between the culture method and the Wood’s lamp in the diagnosis of cats showing clinical symptoms (*P *= 0.163). A statistically significant difference between the culture method and the Wood’s lamp was observed in cats that did not show any clinical symptoms (*P* = 0.005). The sensitivity and specificity of Wood’s lamp examination were 71% and 92%, respectively.

## DISCUSSION

The prevalence of *Microsporum canis* was found to be 64.29% in the shelter. No other species of dermatophytes were detected. *M.* *canis*, the most common cause of dermatophytosis in cats, with a prevalence of up to 90%, is also reported by Svoboda et al. (2008) and Silver (2011). Dermatophyte fungi are a major problem in shelters (Naceradska and Lany 2015). Svoboda et al. (2008) mentioned that dermatophytic fungi occur more frequently in cats than in dogs. It is an important zoonosis (Svoboda et al. 2008; Santana et al. 2018). The occurrence of dermatophyte fungi is often associated with a large financial burden (Pal and Mahendra 2017), which can be devastating for shelters.

The Wood lamp showed relatively good efficiency in our study. The sensitivity of the Wood’s lamp examination was 71%, and the specificity was 92%, which are better results than those reported by DeTar et al. (2019). In their study, the sensitivity of the Wood’s lamp was 66.8% and the specificity was 74.8%; however, the Wood’s lamp was compared with the DTM proof. In our study, we compared the Wood’s lamp and cultivation on SA. Svoboda et al. (2008) no longer generally recommend the use of the Wood’s lamp in the diagnosis of dermatophytes because of frequent false negative or false positive results and also because only *M.* *canis* fluoresces under the Wood’s lamp. On the contrary, Moriello et al. (2017) reported that false negative or positive results are often caused by an inadequate procedure, equipment, uncooperative patient, or lack of examiner’s knowledge and *M.* *canis* shows fluorescence in most cases. However, only *M.* *canis* was identified in the cats we examined*.* The use of the Wood’s lamp could be particularly useful in shelters, which often do not have sufficient funds and therefore do not carry out any diagnostics. The acquisition of the Wood’s lamp and its subsequent use could therefore be a suitable alternative. This statement is supported by Silver (2011), who states that the advantages of the Wood’s lamp are financial savings, ease of use, time-saving and the possibility to examine a large number of animals at once. Vodricka (2005) states that the use of the Wood’s lamp has its benefits, so this method is still used in his opinion, especially in combination with other diagnostic methods. The Wood’s lamp as a tool for rapid initial examination is also mentioned by Marsella (2021). Kiratiwongwan et al. (2022) even mention a handheld UV lamp as a good diagnostic tool. The examination of cats in our work was carried out using the Wood’s lamp and culture on Sabourad agar. Moriello and DeBoer (1991) consider the culture on SA to be the only reliable method; Svoboda et al. (2008) and Pocta (2010) also recommend the culture because it is the only method for reliable diagnosis of dermatophytes and for the determination of relevant species. Moskaluk and VandeWoude (2022) state that culture is the golden standard despite the development of other diagnostic methods.

All cats were sampled for culture by grooming with a sterile toothbrush. The samples were cultured at 25 °C for 21 days and the grown colonies were then examined microscopically. The sampling method of grooming is also recommended by Moriello (2014), however, he recommends the culture at 23 °C, this temperature allegedly being the one at which mould sporulation occurs best. On the contrary, in the study conducted by Fraga et al. (2017), a temperature ranging from 25 °C to 27 °C was used and the sampling method was the same as in our study. Santana et al. (2020) and Taylor (2010) also reported the same method in their studies. Sampling by grooming using a toothbrush and culturing at 25 °C for 21 days is considered reliable (Konvalinova and Mrazkova 2021). Diagnostics using Wood’s lamp took place in a darkened room. The Wood’s lamp was left on for approximately 5 min to warm up and stabilize the wavelength of the emitted radiation, which is also recommended by Taylor (2010) and Silver (2011). On the contrary, Moriello (2019) states that it is not necessary to leave the Wood’s lamp warmed up, but it is necessary to wait for a few minutes to allow the examiner’s eyes to adjust so that the examination can proceed with the greatest possible accuracy.

Statistical evaluation of the results of our work did not reveal any statistically significant difference between the use of the Wood’s lamp and culture on SA in the initial examination of all cats, nor in the group that compared the Wood’s lamp and culture in cats showing clinical symptoms. On the contrary, a statistically significant difference was observed when comparing the Wood’s lamp and culture in cats that did not show any clinical symptoms. Cats can often be asymptomatic carriers (Svoboda and Pospisil 1996). In our conditions, this may be 20% to 30% of cats (Svoboda and Pospisil 1996). Konvalinova and Mrazkova (2021) reported 51.4% of positive cats without clinical symptoms. The sensitivity and specificity determined by us suggest that Wood’s lamp is still quite capable of detecting *M.* *canis.* Its use resulted in a significant capture of positive individuals. Such diagnosed animals could be quarantined at the shelter and treatment could be initiated.

In conclusion, it could be stated that diagnostics of dermatophytic fungi is a relatively large area still under study. The situation around diagnostics in animal shelters is often complicated by a lack of finance, but also by insufficient education and awareness of the staff. Culturing on SA remains one of the most commonly used and at the same time most reliable methods, but it is also a method that can be expensive and time-consuming for some shelters; as an alternative, it is possible to use DTM, where the results are usually known a bit earlier, but the financial burden is still high for large numbers of animals, especially if shelters want to prevent dermatophytosis by testing all new arrivals. The Wood’s lamp can be a pleasant alternative for shelters and a way to reduce or completely prevent the occurrence of dermatophyte fungi. The purchase of Wood’s lamp represents a one-time cost of several thousand Czech crowns and the lamp can serve the shelter for many years. Use of the Wood’s lamp is very easy and quick once the staff is trained. The Wood’s lamp, therefore, appears to be a promising tool that can help shelters to eliminate the occurrence of dermatophyte fungi. It is not possible to recommend the Wood’s lamp in all cases and as the only method, but with well-set-up shelter management and good knowledge of all staff and volunteers, it can be a great help for the shelters, especially when combined with culture methods.
